# Protective evaluation of the commercialized porcine reproductive and respiratory syndrome virus vaccines in piglets challenged by NADC34-like strain

**DOI:** 10.3389/fmicb.2024.1422335

**Published:** 2024-06-26

**Authors:** Zhicheng Liu, Chaosi Li, Yulong Hu, Shuhe Fang, Xiangdong Li, Chunhong Zhang, Lv Huang, Jie Qian, Gang Wang, Aihua Fan, Jianfeng Zhang, Letu Geri

**Affiliations:** ^1^College of Veterinary Medicine, Inner Mongolia Agricultural University, Hohhot, China; ^2^Key Laboratory of Livestock Disease Prevention of Guangdong Province (2023B1212060040), Institute of Animal Health, Guangdong Academy of Agricultural Sciences, Guangzhou, China; ^3^Boehringer Ingelheim Animal Health (Shanghai) Co., Ltd., Shanghai, China; ^4^College of Veterinary Medicine, Yangzhou University, Yangzhou, China; ^5^Shandong Provincial Key Laboratory of Animal Biotechnology and Disease Control and Prevention, College of Veterinary Medicine, Shandong Agricultural University, Taian, China

**Keywords:** PRRSV NADC34-like, VR2332 MLV, R98 MLV, protection efficacy, piglets

## Abstract

In China, the porcine reproductive and respiratory syndrome virus (PRRSV) has undergone several variations over the decades and contributed to the diversity of the clinical epidemic PRRSV strains. This has complicated the prevention and control of PRRS. In particular, the efficacy of the currently available commercial vaccines against the highly pathogenic NADC34-like strains is unclear. Therefore, the objective of this study was to evaluate the protection efficacy of three commercial PRRS modified-live virus (MLV) vaccines derived from classical PRRS VR2332 MLV and R98 MLV against challenge with a heterologous NADC34-like PRRSV strain, JS2021NADC34, which has high pathogenicity in pigs. PRRSV- and antibody-free piglets were immunized with the PRRS VR2332 MLV vaccine or either of two R98 MLV vaccines (from different manufacturers) and were challenged with the JS2021NADC34 strain 28 days after immunization. Rectal temperature, clinical symptoms, viremia and viral shedding from the nose, gross lesions in the thymus and lungs, microscopic lesions and viral distribution in the lungs, as well as the humoral immune response and mortality rates were recorded over a 14-day post-challenge period. The results showed that PRRS VR2332 MLV had better efficacy against the JS2021NADC34 challenge than PRRS R98 MLV, with vaccinated piglets in the former group showing transient and mild symptoms, mild pathological lesions in the lungs, mild thymic atrophy, and low viral levels in sera and nasal swabs, as well as better growth performance and a 100% survival rate. In contrast, two PRRS R98 MLVs exhibited limited efficacy against the JS2021NADC34 challenge, with the piglets in two R98 groups showing obvious clinical symptoms and pathological changes in the lungs and thymus; moreover, there were two deaths caused by PRRS in two R98 groups, respectively. Despite this, the mortality rate was lower than that of the unvaccinated piglets that were challenged with JS2021NADC34. The cumulative results demonstrate that PRRS VR2332 MLV was partly effective against the highly pathogenic PRRSV NADC34-like strain based on the observations over the 14-day post-challenge period. Thus, it might be a viable option among the commercially available vaccines for control of NADC34-like virus infections in swine herds.

## 1 Introduction

Porcine reproductive and respiratory syndrome (PRRS), caused by PRRS virus (PRRSV) infection, has invaded swine farms for nearly 40 years since the first case was reported in 1987 (Keffaber, 1989). PRRSV is comprised of two distinct species, which were designated as *Betaarterivirus europensis* and *Betaarterivirus americense* by the International Committee on Taxonomy of Viruses (ICTV) in 2023 (MSL #39), according to the geographical locations where the initial virus isolations were made. These correspond to the previously identified species PRRSV-1 and PRRSV-2, with nucleotide sequence similarities ranging from 55 to 70% (Lunney et al., 2016). In addition, PRRSV-2 could be further divided into 9 lineages (Shi et al., 2010), and has undergone several variations in China that have specific characteristics, for example, the classical PRRSV, high pathogenic PRRSV, NADC30-like (sublinage 1.8) and NADC34-like (sublinage 1.5) PRRSV, and recombinant strains of various types of PRRSV (Li et al., 2007; Tian et al., 2007; Tong et al., 2007; Zhao et al., 2015; Zhou et al., 2015; Tian, 2017; Zhang et al., 2018). Currently in China, NADC30-like strains is the most dominant strain in swine herds (Yu et al., 2021), NADC34-like PRRSV has drawn great attention for their frequent outbreaks in vaccinated pig herds (Zhang et al., 2018; Liu et al., 2019; Song et al., 2020; Xie et al., 2020; Xu et al., 2020, 2022; Zhao et al., 2022), and the clinical outcomes of infection with NADA30/34-like PRRSV are highly variable, ranging from a lack of obvious symptoms to severe symptoms, including high morbidity and mortality in piglets, respiratory signs in growing pigs, as well as abortion and stillbirth.

Currently, commercial PRRS modified live viruses (MLVs) are being used widely in swine farms in China for dealing with the complex PRRSV situation. These vaccines include the inactivated PRRSV CH-1a strain (lineage 8); Live Chimeric Virus Vaccine (PRRSV PC Strain); the MLVs VR2332 (lineage 5), R98 (lineage 5), and CH-1R (lineage 8) attenuated from classical PRRSV strains; or the MLVs HuN4-F112, TJM-F92, NVDC-JXA1-R, and GDr180 attenuated from highly pathogenic PRRSV strains (lineage 8) (Yu et al., 2021). However, none of these vaccines have been derived from attenuation of the epidemic NADC34-like strains. Moreover, the effects of these MLVs have mainly been demonstrated in response to challenge with homologous PRRSV strains (Tian et al., 2009; Leng et al., 2012; Yu et al., 2015), and very few studies have compared the efficacy of different commercial PRRS MLVs against epidemic NADC34-like strains. Noteworthy, a very recent study on the protective effect of a commercial live attenuated PRRSV vaccine (produced by Yangzhou Uni-Bio Pharmaceutical Co., Ltd.) against NADC34-like strains showed that it could not provide complete protection against the NADC34-like PRRSV infection under experimental conditions based on evaluation of mortality rate, pathological lesions and viral antigens in the lungs and lymphoid tissues, viremia, and PRRSV-specific antibody responses (Yuan et al., 2023).

The present study seeks to contribute to the literature on the clinical prevention and control of PRRS in swine by evaluating the efficacy of commercially available PRRS VR2332 MLV and R98 MLV vaccines against the highly pathogenic heterologous NADC34-like JS2021NADC34 strain of PRRSV.

## 2 Materials and methods

### 2.1 Vaccines and virus

Three commercially available PRRS MLV vaccines, namely, the VR2332 MLV vaccine [Boehringer Ingelheim Animal Health Operations (China) Co., Ltd., Taizhou, Jiangsu, China], the R98-1 MLV vaccine and the R98-2 MLV vaccine from other two different manufactures in China, were studied. The PRRSV strain used for challenge in this study was the PRRSV NADC34-like isolate JS2021NADC34 (GenBank accession number: MZ820388), which belongs to sublineage 1.5 of PRRSV-2 and was passaged three times through porcine alveolar macrophages isolated from the lung tissues of a 4-week-old specific-pathogen-free piglet to obtain a maximum viral titer of 6.46 × 10^6.0^ TCID_50_/mL. The JS2021NADC34 isolate has been shown to be highly pathogenic in weaned piglets and is associated with a 40% mortality rate under experimental conditions (Yuan et al., 2023).

### 2.2 Sequence similarity analysis

The complete genomic sequences, open reading frames (ORFs) 5 and 7, and nonstructural protein (NSP) 2 of the commercial vaccine strains and the JS2021NADC34 isolate used in this trial were aligned by using ClustalW in the BioEdit version 7.1.3.0 (Hall, 1999). The representative PRRSV strains used for sequence similarity analysis are listed in Supplementary Table 1 and Supplementary Figure 1.

### 2.3 Experimental design

Forty-five 21-day-old, castrated piglets that were negative for porcine reproductive and respiratory syndrome virus (PRRSV) were acquired from a local farm free of PRRSV. These piglets were crossbreeds of Landrace and Large White strains [Tianzhong (Shaoguan City) Animal Husbandry Technology Co., Ltd., Shaoguan, Guangdong, China]. The VetMAX^™^ NA and EU PRRSV Reagents (Life Technologies Corporation, Carlsbad, California, United States) and the IDEXX PRRS 2XR test kit (IDEXX Laboratories, Westbrook, Maine, United States) were used to confirm that the experimental animals were free of PRRS. After 1 week of acclimation, based on their weight (Supplementary Figure 2), the animals were randomly divided into the following groups: PRRS VR2332 MLV vaccine group (VR2332, *n* = 10), PRRS R98-1 MLV vaccine group (R98-1, *n* = 10), PRRS R98-2 MLV group (R98-2, *n* = 10), unvaccinated positive control (PC) group (*n* = 10), and unvaccinated negative control or strict control (SC) group (*n* = 5). On the third day after grouping, piglets in the vaccination groups were intramuscularly administered one dose of the PRRS VR2332 MLV vaccine (≥10^4.8^TCID_50_/dose), R98-1 MLV vaccine (≥10^5.0^TCID_50_/dose), or R98-2 MLV vaccine (≥10^5.0^TCID_50_/dose) diluted in a vaccine diluent on the left side of the neck on day 0. Animals in both the unvaccinated control groups received PBS on the same day. At 28 days post immunization (0 days post challenge, 0 dpc), piglets in four groups (the three vaccinated groups and the PC group) were challenged nasally by JS2021NADC34 (0.5 mL per nostril) and also intramuscularly administered JS2021NADC34 (1 mL) at a dose of 3 × 10^5^ TCID_50_/mL. Piglets in the unvaccinated SC group received PBS.

The piglets were monitored daily by measurement of their rectal temperature and assessment of their clinical signs prior to feeding, as described previously (Li et al., 2022). In addition, average daily weight gain (ADWG) during vaccination and challenge in each group was calculated based on the weight of each pig at −31, −1, and 14 dpc. All piglets were euthanized at 14 dpc. All piglets were euthanized under deep anesthesia by intravenously injected with 4 mg/kg·bw of Zoletil^®^ 50 (Tiletamine Hydrochloride and Zolazepam Hydrochloride for Injection, Virbac S.A., Carros, France) at 14 dpc, followed by rapid necropsy. During necropsy, macroscopic lesions in the thymus and lungs were recorded. The macroscopic lung lesions were scored as previously described (Li et al., 2022). In order to further quantify the thymic lesions (atrophy), the relative size of the thymus in the SC group was set at 5 points, with every 20% decrease corresponding to 1 point. Accordingly, complete atrophy (100%) was scored as 0 points. Further, a score of 5 corresponded to a decrease of 0%; a score of 4, a 20% decrease; 3, 40%; 2, 60%; 1, 80%; 0, 100%.

### 2.4 Measurement of viral load in the sera, nasal swabs, and lung tissues by quantitative real-time RT-PCR

Total RNA was extracted using DNA/RNA extraction kits (TianLong Science and Technology Co., Ltd., Xi’an, Shanxi, China) according to the manufacturer’s instructions. Viral load was quantified by TaqMan fluorescent quantitative RT-PCR (RT-qPCR) using the VetMAX^™^ NA and EU PRRSV reagents (Life Technologies Corporation, Carlsbad, California, United States). Serum samples collected every week after vaccination were used to detect CT values for evaluation of viremia caused by vaccines. After challenge, serum samples and nasal swabs collected every 2 days and lung samples obtained during necropsy were analyzed. Nasal swabs were used to measure the magnitude of viral shedding based on calculation of CT values, and serum samples were used to detect the level of viremia based on the TCID_50_ value after challenge. A PRRSV virus stock (JS2021NADC34) with a known titer of 5.2 log_10_ TCID_50_/mL was 10-fold serially diluted into virus-negative 1,640 medium to create dilutions ranging from 10^0^ to 10^−7^ that correspond to theoretical infectious titers of 5.2 to −1.8 log_10_ TCID_50_/mL, which were used to generate a standard curve:


$$CT\;\mathrm{value}=-3.2555\times \log_{10}\;\left({\mathrm{TCID}}_{50}/\mathrm{reaction}\right)+34.62\text{;}$$

$$r^{2}\;\left(\mathrm{correlation}\;\mathrm{coefficient}\right):1.00\text{.}$$

The infection rate of the strain used for challenge was confirmed in serum samples and nasal swabs collected at 2 dpc by using ORF5 sequencing as described previously (Sun et al., 2018).

### 2.5 Serological analysis

Serum antibodies against the PRRSV were detected using the iELISA kit IDEXX PRRS 2XR (IDEXX Laboratories, Westbrook, Maine, United States). A relative S/P index greater than 0.4 was considered positive. Serological analysis was performed on a weekly basis before challenge (from −31 to −1 dpc), and every 2 days after challenge (from 2 to 14 dpc).

Virus neutralization test against the challenge virus (JS2021NADC34) was performed on serum samples as described previously (Li et al., 2022). Briefly, serum samples were heat-inactivated at 56°C for 30 min and serially diluted, and 250 μL each of the diluted serum solutions was mixed with an equal volume of JS2021NADC34 containing 200 TCID_50_. Each mixture was transferred to porcine alveolar macrophage monolayers in 96-well plates and incubated for 72 h at 37°C in an incubator containing 5% CO_2_. The cells were examined for cytopathic effects based on the end-point titers.

### 2.6 Histological examination and immunohistochemistry analysis

Lung tissue samples collected during necropsy after challenge were fixed in 10% neutrally buffered formalin, embedded in paraffin, and cut into 4 μm sections that were used for histopathologic examination following hematoxylin and eosin (H&E) staining and PRRSV antigen detection by immunohistochemistry (IHC). Histopathologic changes in the lung were scored as previously described (Guo et al., 2013). PRRSV was detected using the monoclonal antibody H5D1 (gifted by Professor Huinuan Wang of Jinzhou Medical University) against the PRRSV glycoprotein 5 (GP5) protein by the IHC method according to standard procedures.

IHC staining for PRRSV positivity was scored as follows based on immune markers: 0: no positive signals; 1 mildly scattered positive signals (1 to 20 cells in the entire section); 2 moderately scattered positive signals (less than or equal to 50% of a high magnification field containing immune markers); 3 large numbers of scattered positive signals (more than 50% of high-power fields containing markers and/or at least two to three views containing 30 or more stained cells).

### 2.7 Statistical analysis

The numerical data are expressed as the mean ± S.D. and were analyzed using the GraphPad Prism software (version 9 for Windows; GraphPad Software Inc., Boston, Massachusetts, United States). Differences between groups were assessed using ANOVA followed by the Tukey’s *t*-test. Statistical differences were regarded as significant if *p* < 0.05.

## 3 Results

Except for one piglet in the R98-1 group that died of a bacterial infection at 6 days after vaccination (−22 dpc), none of the piglets exhibited clinical symptoms following immunization with the MLV vaccines. Therefore, the data for the R98-1 group are based on measurements for the remaining nine piglets.

### 3.1 Antibody responses, rectal temperatures, and ADWG following vaccination

**Figure 1:**
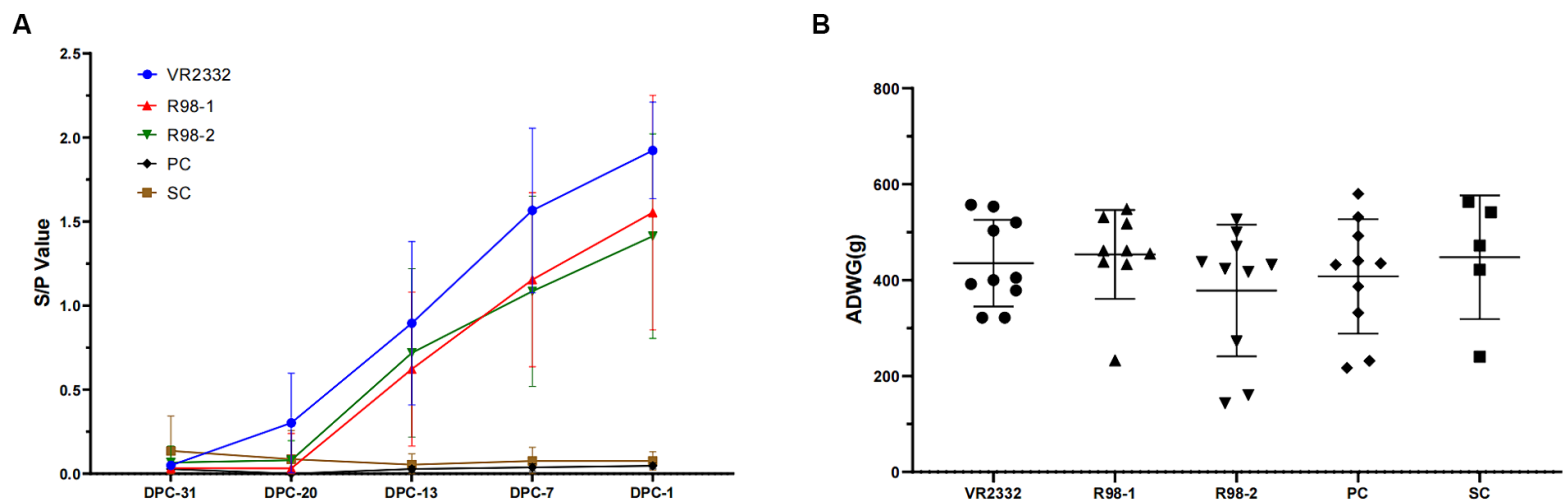
Detection of antibodies against PRRSV and ADWG during immunization. Serum samples were detected for anti-PRRSV antibodies with the IDEXX PRRS 2XR test kit. An S/P ratio equal to or greater than 0.4 was considered positive **(A)**, and ADWG was calculated based on the body weight of piglets at −31 dpc and − 1 dpc **(B)**. Each point in **A** and each line in **B** represent the mean ± S.D. value generated from all piglets in each group.

The humoral immune response induced by the three PRRS MLV vaccines was assessed in sequential serum samples collected from all the experimental piglets (Figure 1A). In the PRRS VR2332 MLV-vaccinated and JS2021NADC34-challenged group, seroconversion occurred after 8 days of vaccination (−20 dpc), and 8 of the 10 piglets developed anti-PRRSV antibodies (S/P > 0.4) after 15 days of vaccination (−13 dpc, Supplementary Figure 3), with the S/P value reaching 1.9 on −1 dpc. In the R98-1-vaccinated and JS2021NADC34-challenged group, 6 of 9 the piglets exhibited seroconversion at −13 dpc (Supplementary Figure 3), with the S/P value reaching 1.6 on −1 dpc. In the R98-2 group, 7 of the 10 piglets developed anti-PRRSV antibodies on −13 dpc (Supplementary Figure 3), with the S/P value reaching 1.4 on −1 dpc. Body weight gain and rectal temperatures were not affected by immunization with any of the vaccines (Figure 1B; Supplementary Figure 4A).

### 3.2 Clinical presentation

**Figure 2:**
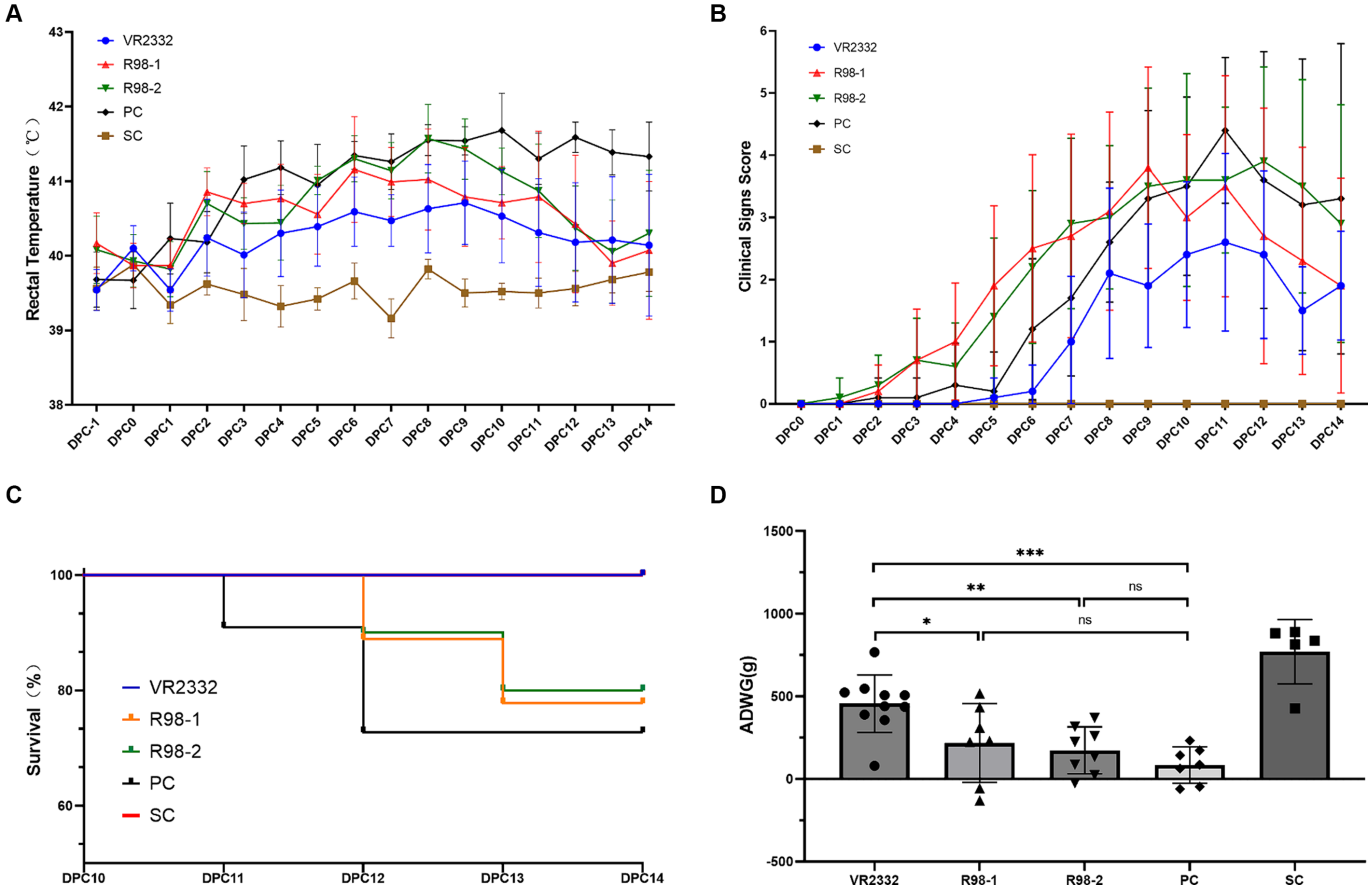
Clinical presentations after the PRRSV JS2021NADC34 challenge. Mean ± S.D. values of the measured rectal temperatures are shown **(A)**. Scores for the clinical signs were evaluated every day before feeding after the JS2021NADC34 challenge **(B)**. Percentage survival of piglets from the five experimental groups is shown **(C)**. ADWG was calculated based on the body weight of piglets at −1 to 14 dpc **(D)**. ^*^p < 0.05, ^**^p < 0.01, and ^***^p < 0.001, and ns indicates no significant difference.

The clinical outcomes were monitored during vaccination and JS2021NADC34 challenge. After infection with JS2021NADC34, the piglets in the PC group developed PRRS symptoms, including anhelation, lethargy, anorexia, and “blue ear” from 6 to 14 dpc, and the clinical scores ranged from 1 to 5 (Figure 2B). Rectal temperature was elevated at 41.0°C at 3 dpc. The high fever was maintained from 3 to 14 dpc, and it peaked at 41.7°C at 10 dpc (Figure 2A). One piglet and two piglets presented with extreme lethargy and were humanely euthanized at 11 and 12 dpc, respectively (Figure 2C). Following JS2021NADC34 challenge, piglets in the VR2332 group showed transient anhelation from 7 to 14 dpc, with the score ranging from 1 to 3 from 7 to 14 dpc (Figure 2B). The piglets had mild fever (from 40.2°C to 40.7°C) from 2 to 11 dpc (Figure 2A). Piglets in the R98-1 and R98-2 groups presented with obvious PRRS clinical signs, including anhelation, lethargy, anorexia, and “blue ear.” In the R98-1 group, piglets had elevated rectal temperature (from 40.6°C to 41.1°C) from 2 to 12 dpc (Figure 2A), with the clinical scores ranging from 1 to 4 from 3 to 14 dpc (Figure 2B). One piglet each presenting with extreme lethargy was humanely euthanized at 12 and 13 dpc (Figure 2C). Similarly, piglets in the R98-2 group also had high fever (from 41.0°C to 41.6°C) from 5 to 10 dpc (Figure 2A), with the clinical scores ranging from 1 to 4 from 5 to 14 dpc (Figure 2B). Additionally, one piglet each presented with extreme lethargy and was humanely euthanized at 12 and 13 dpc (Figure 2C). The SC group piglets had normal rectal temperature for the entire duration of the experimental period. During the viral infection stage, piglets in the four infected groups showed lower weight than piglets from the SC group at 14 dpc. However, before euthanasia, the ADWG of the VR2332 group was significantly higher than that of the other three challenged groups (Figure 2D).

### 3.3 Viremia level and viral shedding in the respiratory tract of infected piglets

Viremia appeared 1 week after vaccination (−20 dpc, Supplementary Figure 4B). Viremia can still be detectable in 10, 8, and 7 piglets (Supplementary Figure 5A), and viral shedding through the nostril was detectable in 4, 1, and 1 piglets (Supplementary Figure 5B) from the VR2332, R98-1, and R98-2 groups at −1 dpc, respectively, and all 39 serum samples and 39 nasal swabs collected at 2 dpc were positive for JS2021NADC34, as verified by ORF5 sequencing.

**Figure 3:**
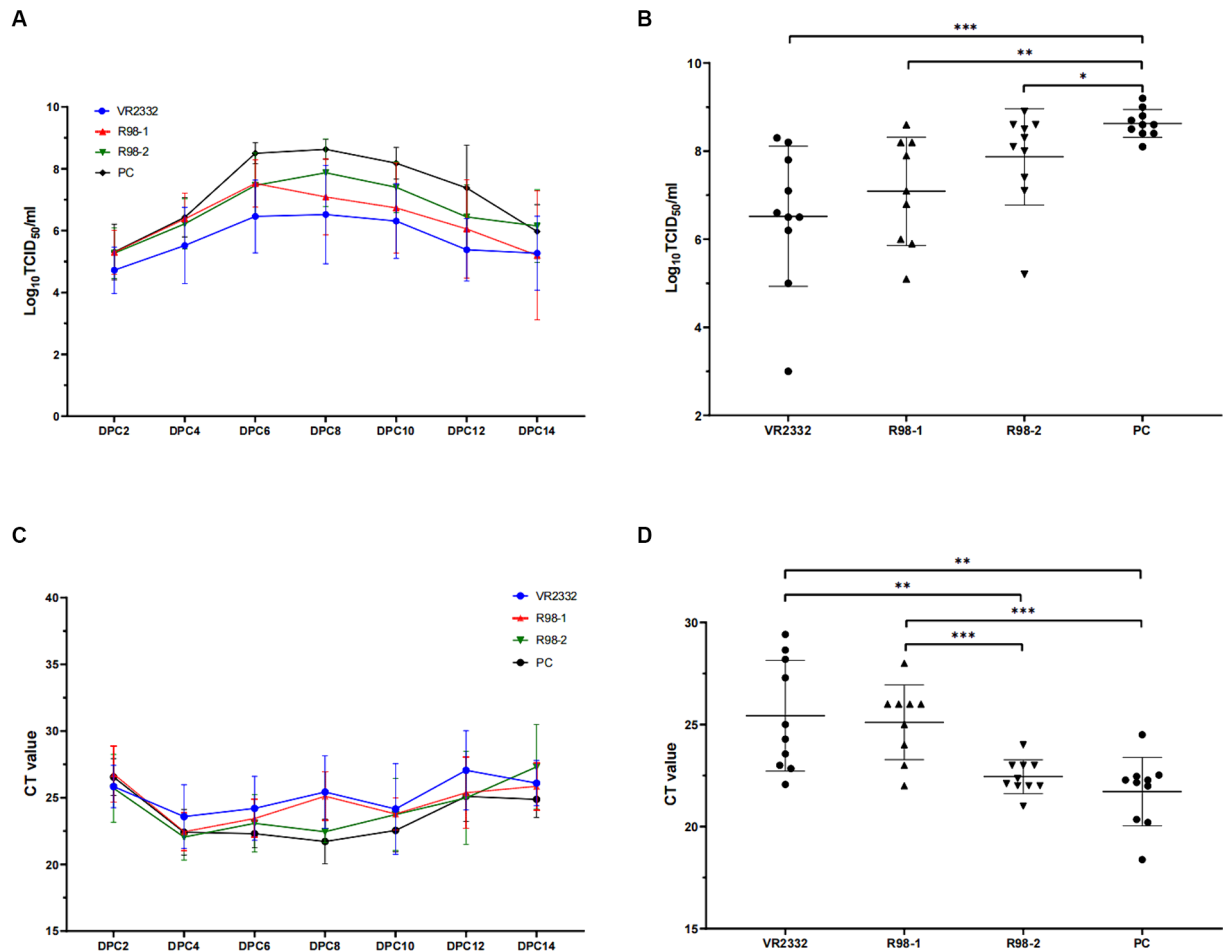
Viremia and detection of viral shedding in piglets. Viral load in serum samples depicted for all measurement days after challenge **(A)** and at 8 dpc **(B)**. Viral shedding in nasal swabs depicted for all measurement days after challenge **(C)** and at 8 dpc **(D)**. ^*^p < 0.05, ^**^p < 0.01, and ^***^p < 0.001.

As shown in Figure 3A, viremia appeared in the JS2021NADC34-infected piglets (PC group) at 2 dpc with an average infectious titer of 10^5.3^ TCID_50_/mL. This peaked at 10^8.6^ TCID_50_/mL at 8 dpc and then decreased gradually till it reached 10^6.0^ TCID_50_/mL at the end of the experiment, that is, at 14 dpc. Piglets in the VR2332 group presented with viremia at 2 dpc based on an average infectious titer of 10^4.7^ TCID_50_/mL, the highest viral load was 10^6.5^ TCID_50_/mL at 8 dpc, and it decreased gradually to 10^5.3^ TCID_50_/mL at 14 dpc. For the R98-1 group, viremia developed at 2 dpc based on an average infectious titer of 10^5.3^ TCID_50_/mL that peaked to 10^7.5^ TCID_50_/mL at 6 dpc and then decreased to 10^5.2^ TCID_50_/mL at 14 dpc. Similarly, piglets in the R98-2 group developed viremia at 2 dpc based on an average infectious titer of 10^5.3^ TCID_50_/mL that peaked to 10^7.9^ TCID_50_/mL at 8 dpc and then decreased to 10^6.2^ TCID_50_/mL at 14 dpc. At 8 dpc (Figure 3B), the viral load in the serum samples from the VR2332, R98-1, and R98-2 group was significantly lower than that in the PC group. No virus was detected in the SC group throughout the study period.

Nasal swab samples were used to detect viral shedding in piglets during the JS2021NADC34 challenge. All the swabs were positive for PRRSV from 2 dpc based on a CT value of ~26, and the nasal swab samples remained positive till the end of the experiment, at 14 dpc (Figure 3C). At 8 dpc, the viral load in the swabs from the VR2332 and R98-1 group was significantly lower than that in the swabs from the R98-2 and PC group (Figure 3D).

### 3.4 Macroscopic lesions in the thymus and lungs

**Figure 4:**
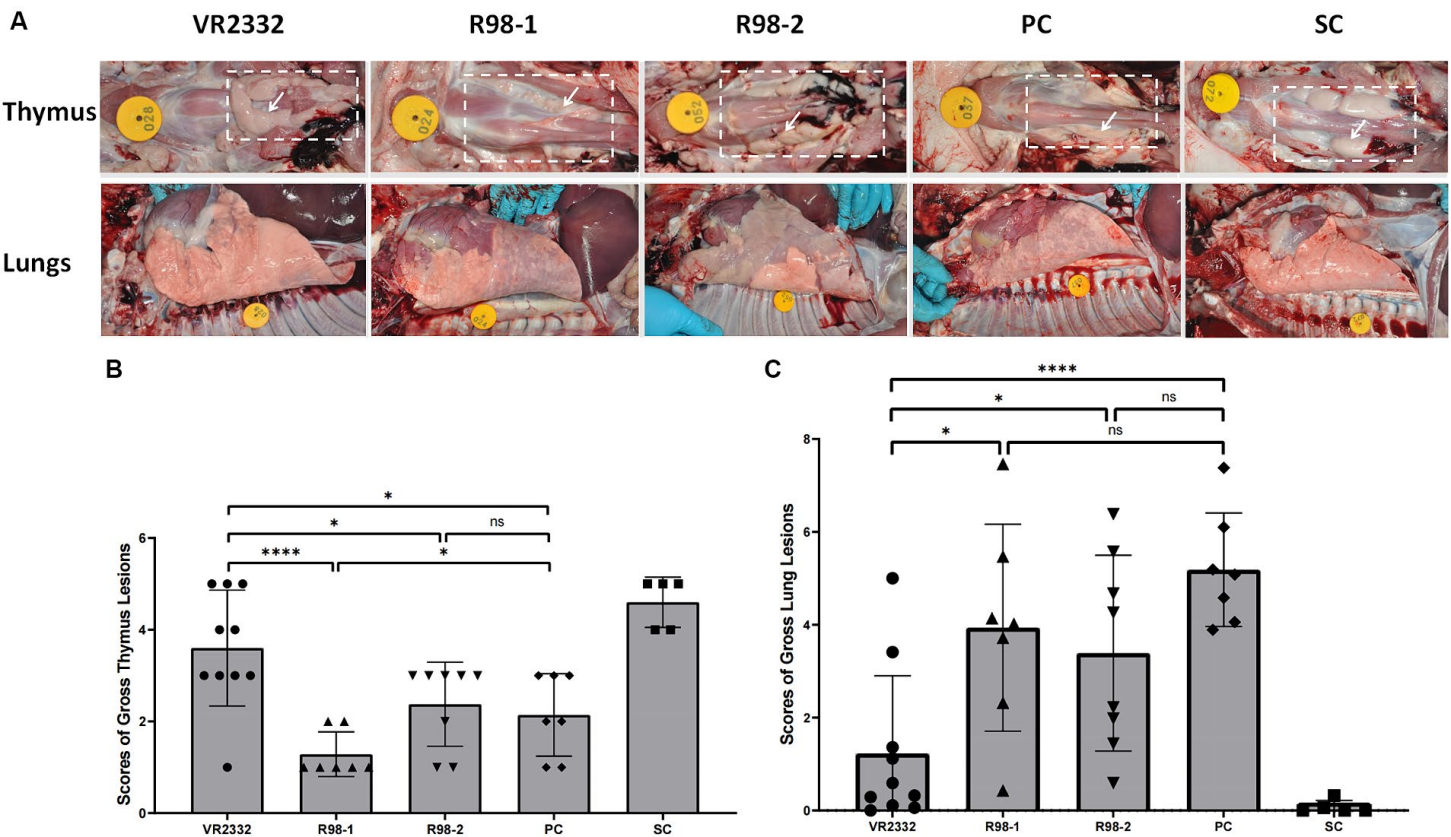
Macroscopic lesions in the thymus and lungs during necropsy at 14 dpc. Representative samples of the thymus and lungs from one piglet in each group **(A)**. The white dashed box denotes the area where the thymus is located, and the white arrow denotes the representative thymus. Mean ± S.D. gross thymus lesion scores **(B)** and lung lesion scores **(C)** for each group are shown. ^*^p < 0.05, ^**^p < 0.01, ^***^p < 0.001, and ^****^p < 0.0001, and ns indicates no significant difference.

At 14 dpc, 10, 7, 8, and 7 pigs survived in the VR2332, R98-1, R98-2, and PC groups, respectively (Figure 2C). Macroscopic lesions in the thymus and lungs were recorded during necropsy at 14 dpc (Figure 4). Out of the 10 piglets in the VR2332 group, three showed no obvious thymic lesion or atrophy, six presented with mild thymic atrophy, and only one developed severe thymic atrophy (average atrophy score = 3.6) (Figure 4B). Corresponding results were obtained for interstitial pneumonia in the apical or cardiac lobes of the lung (Figure 4A), with the average score for lung lesions being 1.2 (Figure 4C). All the seven piglets in the R98-1 group that were necropsied had severe thymic atrophy at 14 dpc (average atrophy score = 1.3) (Figure 4B) and signs of interstitial pneumonia in the lobes of the lung (average lung lesion score = 3.9) (Figures 4A,C). Similarly, all eight piglets in the R98-2 group that were necropsied had obvious thymic atrophy at 14 dpc (average atrophy score = 2.4) (Figure 4B) and interstitial pneumonia in all lobes of the lung (average lung lesion score = 3.4) (Figures 4A,C). In the PC group, both severe thymic atrophy and interstitial pneumonia were observed in thymus and lungs (Figure 4A), with thymus atrophy and lung lesions scores of 2.1 and 5.2, respectively (Figures 4B,C). No thymic atrophy or interstitial pneumonia was observed in piglets from the SC group during necropsy.

### 3.5 Histopathological lesions and distribution of the virus in the lungs

**Figure 5:**
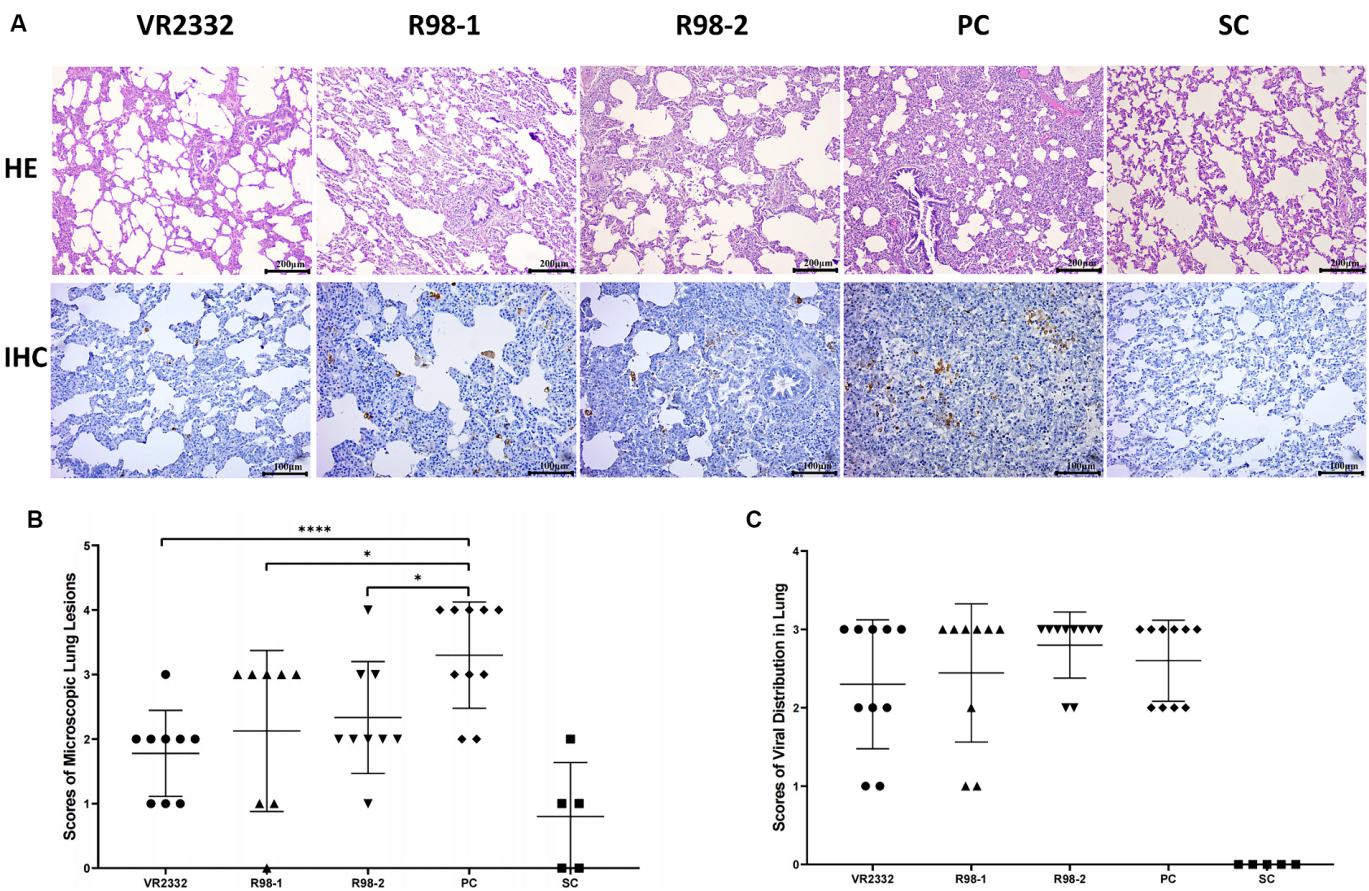
Histopathological lesions and viral distribution in necropsied lungs. Representative samples of the lung from one piglet of each group are shown **(A)**. The mean ± S.D. microscopic lung lesion scores **(B)** and viral distribution scores **(C)** for each group are shown. ^*^p < 0.05, ^**^p < 0.01, ^***^p < 0.001, and ^****^p < 0.0001.

To better characterize the differences in the lung lesions induced by JS2021NADC34 infection in different groups, the lungs were examined at a microscopic level for signs of histopathological damage. It was not possible to determine the microscopic pathological scores for interstitial pneumonia caused by secondary bacterial infections in any of the groups. For the scoring of lesions, 9, 8, 9, and 9 lungs were included from the VR2332, R98-1, R98-2, and PC groups, respectively. In the piglets from the VR2332 group, slight alveolar wall widening with inflammatory cell infiltration was observed (Figure 5A), and the average lung lesion score was 1.8 (Figure 5B). In contrast, piglets from the R98-1 and R98-2 groups showed alveolar miniaturization with severe interstitial pneumonia lesions and massive inflammatory cell infiltration, with scores of 2.1 and 2.3, respectively (Figures 5A,B); in addition, a large number of neutrophils were observed in the alveolar region of the R98-2 group piglets (Figure 5A). In the PC group piglets, the alveoli were almost invisible, and suppurative pneumonia and severe diffuse interstitial proliferation of small lymphocytes were observed (Figure 5A), with a score of 3.3 (Figure 5B). The lungs from SC group piglets had normal features.

Viral GP5 antigen was detected by IHC in the lung of experimental piglets using the monoclonal antibody H5D1, but no significant differences were observed among the JS2021NADC34-challenged groups. A few GP5-positive cells were detected in the lungs of piglets from the VR2332 group and R98-1 group (Figure 5A), with IHC staining scores of 2.3 and 2.4, respectively (Figure 5C). These scores were lower than those in the R98-2 group and PC group, which had scores of 2.8 and 2.6, respectively (Figure 5C). No viral antigen was detectable in lung samples from the SC group.

### 3.6 Detection of virus in the lungs and antibodies against the PRRSV in the sera

**Figure 6:**
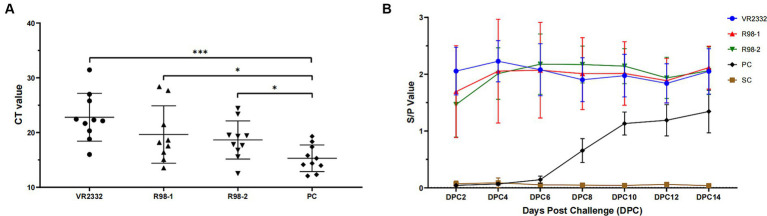
Detection of virus in the lungs and antibodies against the PRRSV in sera. CT values indicating the viral load in the lungs of experimental piglets are shown **(A)**. The anti-PRRSV antibody titer is indicated by the S/P ratio, with a value of 0.4 or higher than 0.4 considered positive **(B)**. ^*^p < 0.05, ^**^p < 0.01, and ^***^p < 0.001.

The viral load in the lungs of piglets from each group was quantified by TaqMan fluorescent RT-qPCR during 14 dpc. The PRRSV genome was detected in the lungs of all piglets infected with JS2021NADC34, but the viral load differed among groups: the average CT value for the VR2332 group, R98-1 group, R98-2 group, and PC group was 22.8, 19.7, 18.6, and 15.3, respectively (Figure 6A).

To better understand the humoral immune response elicited by vaccination and challenge with JS2021NADC34, the anti-PRRSV antibody titer was detected using an iELISA kit (Figure 6B). Piglets from the three immunized groups developed high titers at 30 days after immunization (2 dpc), and the JS2021NADC34 challenge resulted in a slight increase in the titer. The high titers were maintained till the end of the experiment, at 14 dpc. In the PC group, seroconversion occurred at 6–8 dpc and increased quickly till 10 dpc. The average S/P value reached up to 1.13 at 10 dpc and increased further to 1.34 at 14 dpc. No anti-PRRSV antibodies were detected in piglets from the SC group during the duration of the experiment.

Neutralizing antibodies (NAs) were not detectable (NA titer <1:2) in any sera in any pigs of the groups before and after challenge.

## 4 Discussion

The current study tried to determine whether the classical PRRS MLV vaccines can offer protection against NADC34-like clinical epidemic strains by testing whether immunization of piglets with three of these vaccines, the PRRS VR2332 MLV vaccine and two R98 MLV vaccines, could offer protection against the highly pathogenic JS2021NADC34 isolate.

First, we determined the nucleotide homology of WGS, *ORF5*, *ORF7*, and *NSP2* between JS2021NADC34 (sublineage 1.5) and the three vaccine strains (VR2332 MLV, R98-1, and R98-2 MLVs; lineage 5) and found that it was 82.0–82.1%, 86.0–87.2, 89.8, and 67.0%, respectively (Supplementary Table 1). Aligned with JS2021NADC34 strain, we discovered 30 and 31 differential amino acid residues in glycoprotein 5 (GP5) (the major envelope protein encoded by *ORF5*) of VR2332 MLV and two R98 MLVs, respectively; also 14 in nucleocapsid (N) protein encoded by *ORF7* (Supplementary Figures 1A,B). JS2021NADC34 NSP2 has a unique continuous 100-aa deletion corresponding to positions 328–427 in the NSP2 protein of VR2332 MLV and two R98 MLVs (Supplementary Figure 1C). Based on the WGS, *ORF5*, *ORF7*, and *NSP2*, the divergence in nucleotide sequences between VR2332 and the two R98 vaccine strains were found to be 1, 0.3, 0, and 0%, respectively (Supplementary Table 1). In the GP5 protein, both VR2332 and the two R98 vaccine strains exhibit variation at one antigenic neutralization site (amino acid 33, Supplementary Figure 1A). The VR2332 MLV and both R98 MLVs have in common five amino acid differences within the NSP2 protein, occurring at residues 82, 180, 475, 491, and 636 (Supplementary Figure 1C). Furthermore, amino acid 935 serves as a distinguishing feature for the R98-1 MLV in comparison to the VR2332 MLV and the R98-2 MLV (Supplementary Figure 1C).

During the immunization period, none of the vaccines affected the growth of the pigs, and all three induced good humoral immune response against PRRSV. However, the VR2332 MLV vaccine induced antibodies earlier and at a higher level compared to the other two vaccines (Figure 1). The clinical and histopathological signs and the mortality rate in the non-vaccinated control group (the PC group) after challenge with the JS2021NADC34 isolate were consistent with those reported in previous inoculation experiments (Yuan et al., 2023). In the vaccinated groups, the protective effects of the three vaccines were obviously different. When piglets from the VR2332 group were challenged with JS2021NADC34, they presented with transient clinical symptoms mild fever, low histopathological sign scores, and a low level of viremia over a 14 dpc period. Importantly, their survival rate was 100% over the period. The piglets immunized with the two R98 MLV vaccines showed more severe PRRS signs after challenge, including high rectal temperature and higher histopathological scores. However, the histopathological signs and viremia were milder and survival was better compared to the nonvaccinated challenged (PC) group. Similar protective effects of a commercial live attenuated PRRS vaccine against NADC34-like strains have been reported (Yuan et al., 2023). With regard to changes in body weight, in the immunized groups, a varying degree of weight loss was observed (VR2332 vs. SC, *p* = 0.0072; R98-1 vs. SC, *p* = 0.0017; R98-2 vs. SC, *p* < 0.0001; PC vs. SC, *p* < 0.0001); in fact, the VR2332 MLV vaccine led to significantly higher weight gain than the other two vaccines (VR2332 vs. R98-1, *p* = 0.0307; VR2332 vs. R98-2, *p* = 0.0019; VR2332 vs. PC, *p* = 0.0002). The weight gain associated with the VR2332 MLV vaccine could mean a reduction in economic losses in the field (Haiwick et al., 2018).

Viral load in sera and tissues is an important indicator of the clinical progression and stage and extent of tissue lesions caused by PRRS (Roca et al., 2012; Han et al., 2013; Lager et al., 2014; Wei et al., 2019), and it is also an important indicator for predicting infection pressure in the PRRS environment (Haiwick et al., 2018). Therefore, we detected the viral load of PRRSV in nasal swabs, sera, and lung tissues with RT-qPCR, as well as viral distribution in the lungs through IHC, in order to assess the efficacy of the PRRSV vaccines. There were differences in the viremia level and virus shedding through the respiratory tract after challenge among the four challenge groups that is, the VR2332, R98-1, R98-2, and PC groups, that changed with time over 6 to 12 dpc (Figures 3A,C). These results are consistent with those of previous reports that the VR2332 MLV vaccine decreases the level of viremia and virus shedding during challenge with other PRRSV isolates (Ouyang et al., 2016; Madapong et al., 2020; Li et al., 2022). The viral load of PRRSV and viral distribution in the lung appear to be consistent with the observations in the nasal swab samples: that is, the viral load in the lung was lower that in the VR2332 and R98-1 groups than in the R98-2 and PC groups (VR2332 vs. R98-1, *p* = 0.1730; VR2332 vs. R98-2, *p* = 0.0308; VR2332 vs. PC, *p* = 0.0002; PC vs. R98-1, *p* = 0.0304). It has been confirmed that there is a dose-dependent relationship between production indicators, such as body temperature and average daily weight gain, and infection of PRRSV. Within a certain range, the higher the infection dose, the higher the body temperature, and the smaller the average daily weight gain (Haiwick et al., 2018). Therefore, immunizing pigs with the VR2332 MLV vaccine reduces the time and amount of virus elimination after infection of PRRSV NADC34-like JS2021NADC34 strain, further it could reduce the viral load in the environment and exposure to susceptible animals, as well as could improve production indicators such as average daily weight gain.

The severity of thymic atrophy induced by PRRSV infection is correlated with the degree of immunosuppression and determines whether the host is susceptible to other pathogens (Wang et al., 2020). Previous investigations have shown that PRRSV can induce apoptosis in central and peripheral immune organs, which include, but are not limited to, the thymus, tonsil, inguinal lymph nodes, spleen, and lungs (Wang et al., 2020). The degree of PRRSV-induced thymic atrophy and thymocyte apoptosis has also been found to be dependent on the PRRSV strain (Wang et al., 2020). In our study, the PC group showed severe thymic atrophy in the thymus and interstitial pneumonia in the lungs, which are indicative of the strong pathogenicity of the JS2021NADC34 strain and its ability to induce apoptosis and lesions in the thymus and the lungs, respectively. With regard to the effects of immunization with the commercial vaccines, piglets in the VR2332 group developed only slight thymic atrophy (PC vs. VR2332, *p* = 0.0197) and interstitial pneumonia in partial lobes of the lungs (PC vs. VR2332, *p* < 0.0001), while those in the two R98 groups showed mild to medium thymic atrophy (PC vs. R98-1, *p* = 0.0468; PC vs. R98-2, *p* = 0.6298) and interstitial pneumonia in most of lobes of the lungs (PC vs. R98-1, *p* = 0.2190; PC vs. R98-2, *p* = 0.0699). This finding demonstrates that these three vaccines alleviated the immunosuppression induced by PRRSV to different degrees. Importantly, they reduced the chances of secondary infections by other pathogens.

It should be pointed out that no neutralizing antibodies against the JS2021NADC34 strain were detectable in the sera of any of the experimental groups before or after the challenge. Thus, the PRRS VR2332 MLV and two R98 MLV vaccines may be unable to, or have limited ability to, induce the production of neutralizing antibodies against the heterologous JS2021NADC34 strain, as described in previous reports (Vu et al., 2011; Zhou et al., 2012; Lunney et al., 2016; Yuan et al., 2023). In addition, GP5 is the major inducer of neutralizing antibodies *in vivo* (Ansari et al., 2006), in which the changes of amino acid sequences at two identified neutralizing active epitopes (amino acid position 32–34, and 57–59, Supplementary Figure 1A) may be a possible reason for not producing neutralizing antibodies (Kim et al., 2013). One limitation of this study was that we did not assess innate immune response and cell-mediated immune response, even though varying degrees of immune-related organ lesions, such as thymic atrophy and interstitial pneumonia, were observed. In the future, it would be useful to use different evaluation methods for the study of PRRSV pathogenesis and the protection mechanisms of vaccines.

In conclusion, this study showed that the VR2332 MLV and two R98 MLV vaccines had varying effects on the level of viral load in the sera and lungs, viral shedding from the nose, and thymic and lung lesions in immunized piglets that were exposed to the high pathogenicity PRRSV NADC34-like JS2021NADC34 strain. Overall, compared to the two R98 MLV vaccines (from two manufactures), the VR2332 MLV vaccine had better protective efficacy under the current experimental conditions. Therefore, the classical PRRS MLV vaccines may offer protection to various degrees against the clinical epidemic NADC34-like strain in swine herds.

## Data availability statement

The original contributions presented in the study are included in the article/Supplementary material, further inquiries can be directed to the corresponding authors.

## Ethics statement

The animal study was approved by the Animal Ethics Committee of Institute of Animal Health, Guangdong Academy of Agricultural Sciences (approval number YC-PT2023009). The study was conducted in accordance with the local legislation and institutional requirements.

## Author contributions

ZL: Data curation, Methodology, Writing – original draft, Writing – review & editing. CL: Data curation, Methodology, Writing – original draft, Writing – review & editing. YH: Methodology, Writing – review & editing. SF: Methodology, Validation, Writing – review & editing. XL: Methodology, Resources, Writing – review & editing. CZ: Methodology, Project administration, Writing – review & editing. LH: Methodology, Writing – review & editing. JQ: Conceptualization, Methodology, Writing – review & editing. GW: Methodology, Writing – review & editing. AF: Conceptualization, Methodology, Validation, Writing – review & editing. JZ: Funding acquisition, Methodology, Writing – original draft, Writing – review & editing. LG: Formal analysis, Methodology, Validation, Writing – review & editing.
