# Supplementary material for: Protective evaluation of the commercialized porcine reproductive and respiratory syndrome virus vaccines in piglets challenged by NADC34-like strain
Source: Front Microbiol. 2024 Jun 26;15:1422335. doi: 10.3389/fmicb.2024.1422335 (PMC11233820; doi:10.3389/fmicb.2024.1422335)
Supplement: Supplementary file 1 [file Data_Sheet_1.pdf]

Supplementary Material

Protective evaluation of the commercialized PRRSV vaccines in piglets challenged by NADC34-like strain

Zhicheng Liu [1, 2, †], Chaosi Li [3, †], Yulong Hu [3], Shuhe Fang [3], Xiangdong Li [4], Chunhong Zhang [2], Lv Huang [3], Jie Qian [3], Gang Wang [5], Aihua Fan [3], Jianfeng Zhang [2, *] and Letu Geri [1, *]

[1] College of Veterinary Medicine, Inner Mongolia Agricultural University, Hohhot 010018, China, [2] Key Laboratory of Livestock Disease Prevention of Guangdong Province (2023B1212060040); Institute of Animal Health, Guangdong Academy of Agricultural Sciences, Guangzhou 510640, China, [3] Boehringer Ingelheim Animal Health (Shanghai) Co. Ltd., Shanghai 200040, China, [4] College of Veterinary Medicine, Yangzhou University, Yangzhou 225009, China, [5] Shandong Provincial Key Laboratory of Animal Biotechnology and Disease Control and Prevention, College of Veterinary Medicine, Shandong Agricultural University, Taian 271018, China

[†] These authors contributed equally.

* **Correspondence:** 13668939298@139.com (J.Z.), geriletursy@imau.edu.cn (L.G.)

Supplementary Table 1. Whole genome sequence (WGS), *ORF5*, *ORF7* and *NSP2* nucleotide identities between JS2021NADC34 and vaccine strains

Genome fragments	Isolate	Pairwise % identity		
		**VR2332 MLV**	**R98-1 MLV**	**R98-2 MLV**
WGS	JS2021NADC34	82.1	82.0	82.0
ORF5		87.2	86.9	86.9
ORF7		89.8	89.8	89.8
NSP2		67.0	67.0	67.0

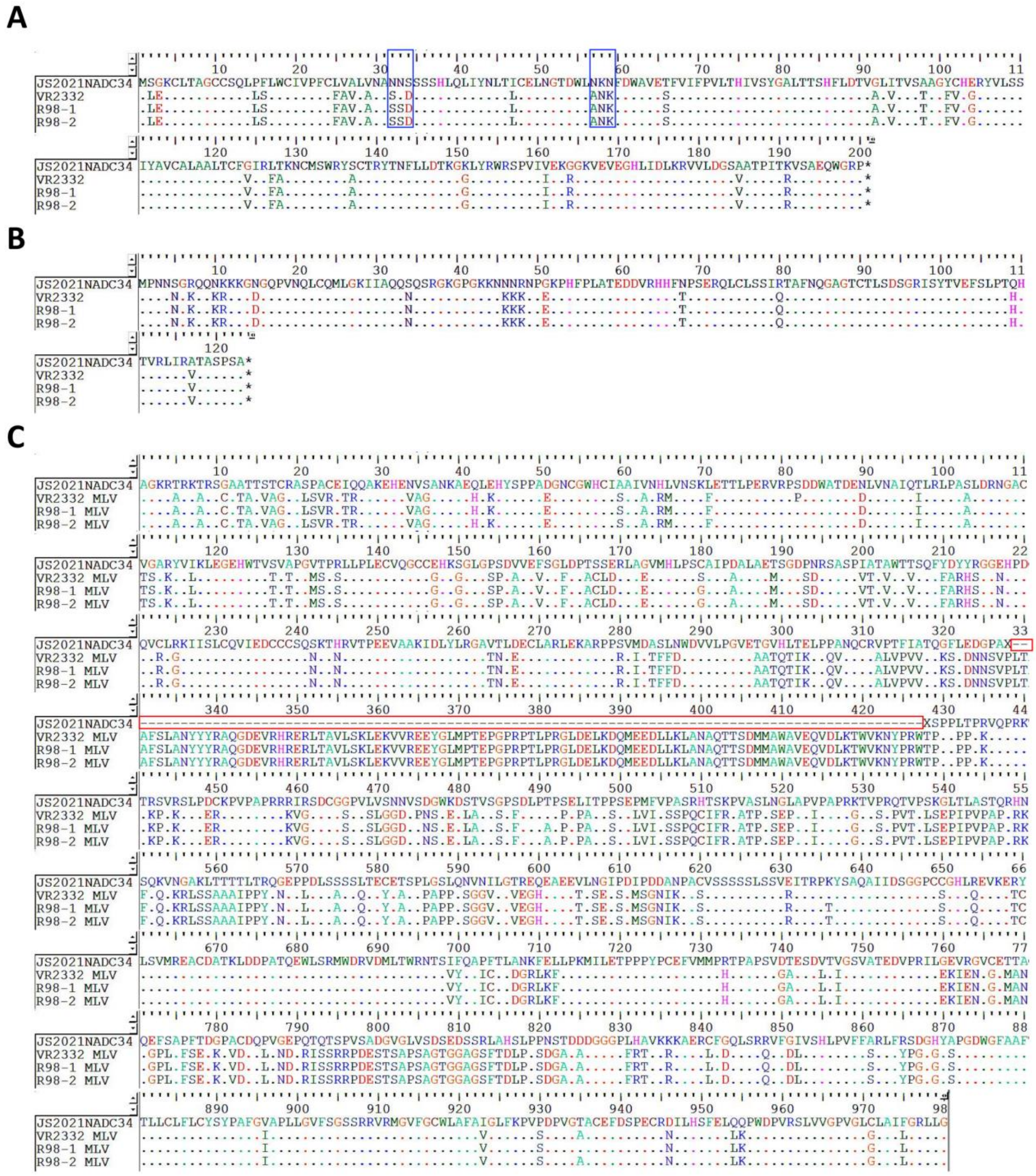

Supplementary Figure 1 The alignment of the amino acid sequence of glycoprotein 5 (GP5) (A), nucleocapsid (N) protein (B), and NSP2 protein (C). GP5, N, and NSP2 proteins were deduced by the *ORF5*, *ORF7*, and *NSP2* genes of JS2021NADC34, VR2332, and two R98 strains, respectively. 30 and 31 differential amino acid residues are existed in the GP5 between JS2021NADC34 strain and VR2332 MLV, two R98 MLVs, respectively; in which, amino acid positions 32-34, and 57-59 (blue box) are identified neutralizing active epitopes. Similarly, 14 differential amino acid residues are existed in N protein. JS2021NADC34 NSP2 has a unique continuous 100-aa deletion (red box) corresponding to amino acid positions 328-427 in the NSP2 protein of VR2332 MLV and two R98 MLVs.

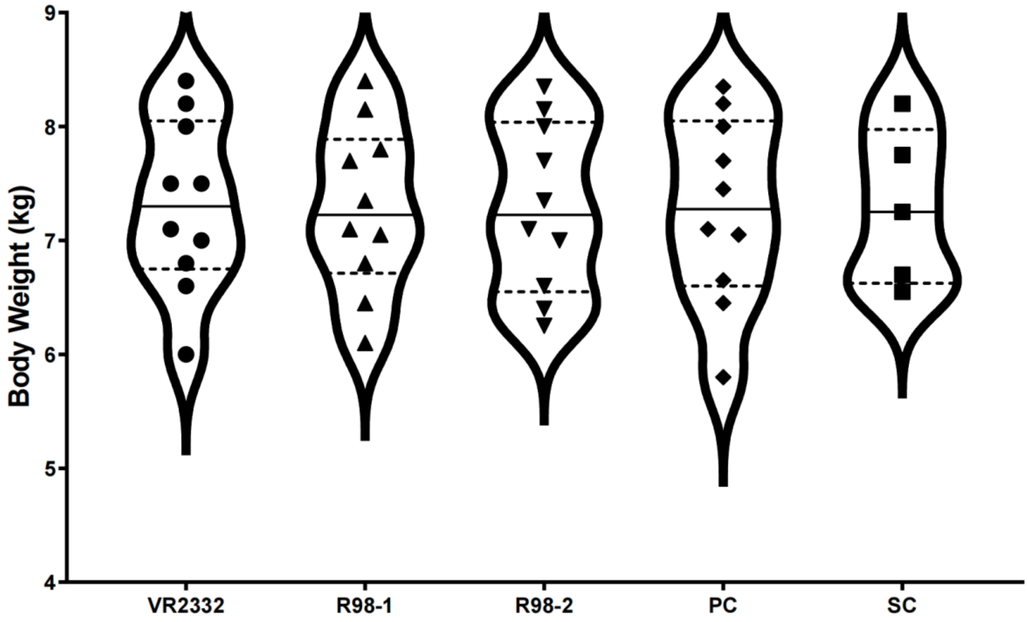

Supplementary Figure 2 Grouping based on the weight of each pig at three days before vaccination (-31 dpc). Forty-five piglets were randomly divided into five groups based on their weight, 10 piglets per group for vaccination and/or viral inoculation, the negative control group has five piglets. The average body weight of each piglet in VR2332, R98-1, R98-2, PC, and SC groups were 7.31, 7.29, 7.29, 7.275, and 7.29 kilogram (kg), respectively.

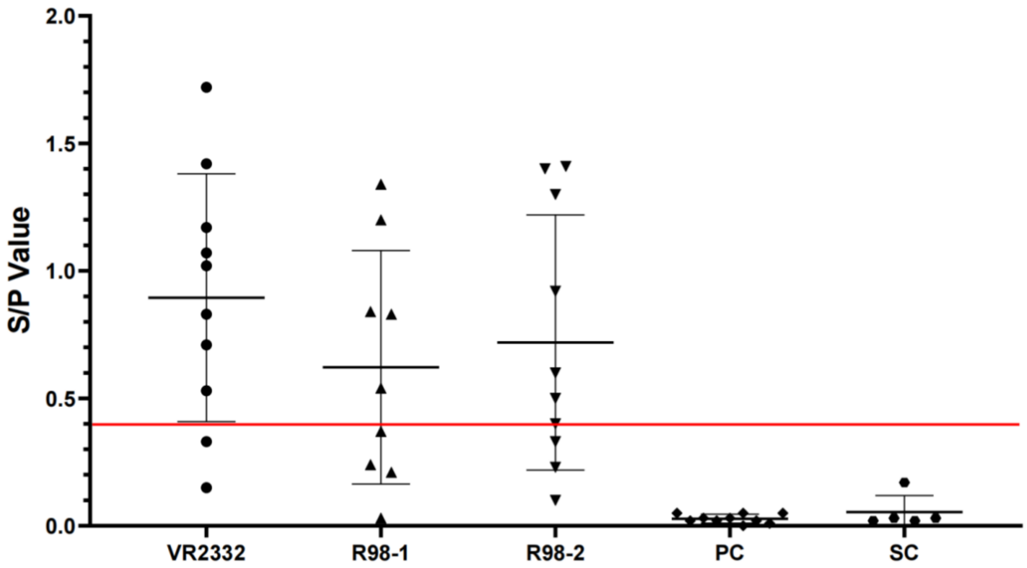

Supplementary Figure 3 Antibodies against PRRSV in serums on 15 days of vaccination (-13 dpc) for each group. The serum samples were detected for anti-PRRSV with IDEXX PRRS 2XR test kit with S/P ratio of 0.4 (red line) or greater considered positive.

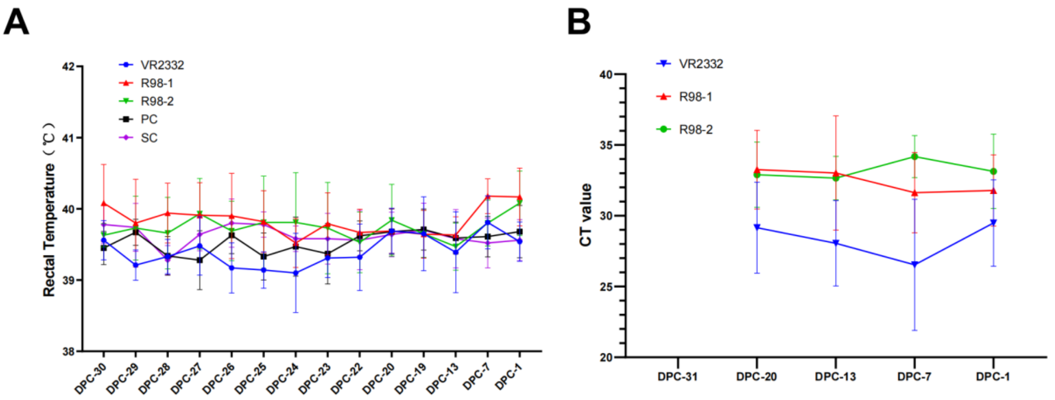

Supplementary Figure 4 Rectal temperatures and viremia after vaccination. Mean ± *SD* values of the measured rectal temperatures for each group after vaccination are shown (A). After vaccination, the rectal temperatures of each pig were measured daily for the first week, and for the remaining three weeks, the rectal temperatures were further measured once a week. Evaluation of viremia caused by vaccines for VR2332, R98-1, R98-2 groups after vaccination (B). Serum samples collected every week after vaccination were used to detect CT values with TaqMan fluorescent quantitative RT-PCR (RT-qPCR) using the VetMAX™ NA and EU PRRSV reagents.

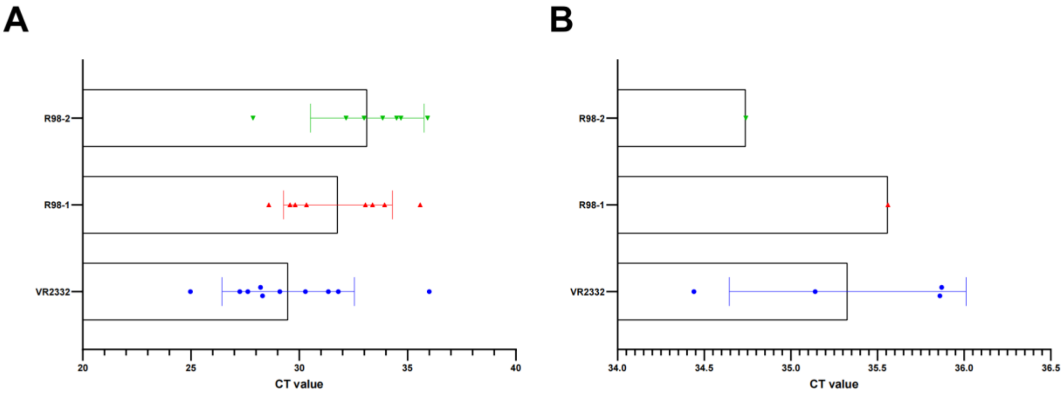

Supplementary Figure 5 Viremia (A) and viral shedding through the nostril (B) of VR2332, R98-1, and R98-2 groups at -1 dpc. Viremia was detectable in 10, 8, and 7 piglets, and viral shedding from the nose was detectable in 4, 1, and 1 piglets from the VR2332, R98-1, and R98-2 groups, respectively.
